# Ampelopsin Induces Cell Growth Inhibition and Apoptosis in Breast Cancer Cells through ROS Generation and Endoplasmic Reticulum Stress Pathway

**DOI:** 10.1371/journal.pone.0089021

**Published:** 2014-02-13

**Authors:** Yong Zhou, Furong Shu, Xinyu Liang, Hui Chang, Linying Shi, Xiaoli Peng, Jundong Zhu, Mantian Mi

**Affiliations:** Research Center for Nutrition and Food Safety, Institute of Military Preventive Medicine, Third Military Medical University, Chongqing Key Laboratory of Nutrition and Food Safety, Research Center for Medical Nutrition, Chongqing, P.R. China; Juntendo University School of Medicine, Japan

## Abstract

Ampelopsin (AMP), a major bioactive constituent of *Ampelopsis grossedentata*, exerts a number of biological effects. In this study, we investigated its anti-cancer activity in human breast cancer cell lines, and explored the underlying mechanism of this action. Our results showed that treatment with AMP dose-dependently inhibited cell viability and induced apoptosis in MCF-7 and MDA-MB-231 breast cancer cells without cytotoxicity in human normal breast epithelial cells MCF-10A. Meanwhile, AMP dose- dependently triggered reactive oxygen species (ROS) generation in both breast cancer cells. The ROS scavenger N-acetyl-L-cysteine (NAC) strongly attenuated AMP-induced ROS production, along with cell growth inhibition and apoptosis. Furthermore, AMP was observed to activate endoplasmic reticulum (ER) stress, as evidenced by the up-regulation of ER stress-related proteins, including GRP78, p-PERK, p-elF2α, cleaved ATF6α and CHOP, while knockdown of ATF6α or PERK markedly down-regulated AMP-induced CHOP expression. Blocking ER stress using 4-phenylbutyric acid not only down-regulated AMP-induced GRP78 and CHOP expression, but also significantly decreased AMP-induced cell growth inhibition and apoptosis, whereas ER stress inducer thapsigargin played opposing effects. Additionally, NAC inhibited AMP-induced ER stress by down-regulating GRP78 and CHOP expression. Conversely, blocking ER stress using CHOP siRNA decreased AMP-induced ROS production and cell apoptosis. Taken together, these results demonstrate that AMP has anti-tumor effects against breast cancer cells through ROS generation and ER stress pathway, which therefore provide experimental evidences for developing AMP as a new therapeutic drug for breast cancer.

## Introduction

Although significant progress has been achieved in the development of targeted therapies, breast cancer as the most common cancer develops to be the leading cause of cancer-related deaths in women [1], [2]. It is estimated that over 1,000,000 women are newly diagnosed with breast cancer every year worldwide, and that more than 400,000 cases will die from breast cancer[2]. New therapeutic strategy and chemotherapeutic candidates for breast cancer are therefore urgently needed to explore[3].

Natural products are major resources of prospective anti-cancer candidates [4], [5], for example paclitaxel, commonly used in breast cancer treatment, is isolated from the bark of the Pacific yew tree [6], [7]. *Ampelopsis grossedentata* is widely distributed in South China, and its tender stems and leaves are used as a healthy tea product. Ampelopsin (AMP), also named dihydromyricetin, one of flavonoids, is the major bioactive constituent of *Ampelopsis grossedentata* [8]–[10]. It has been reported that AMP exerts a number of biological and pharmacological actions including hypoglycemic, anti-oxidative, and hepato-protective effects [11], [12]. Recent studies showed that AMP has potent anti-cancer activities against several cancers, including liver, prostate and bladder [13]–[15]. However, its anti-tumor effects on breast cancer have not been explored and its underlying mechanism of action remains to be elucidated.

As multi-faceted signaling molecules involved in a number of cellular functions, reactive oxygen species (ROS) exert key roles in determination of cell fate-death or survival [16], [17]. Recently, ROS have been identified as potential targets for seeking novel anti-cancer drugs [18], [19]. Numerous investigations suggested that endoplasmic reticulum (ER) stress could be either a cause, or a result, of increased ROS generation [17], [20]. ER stress further activates signaling pathways of promoting cell death, and targeting ER stress response as a new anti-cancer strategy [21], [22]. Thus, the roles of ROS generation and/or ER stress in cell death have attracted extensive attention [3], [23].

In this study, we aimed to investigate whether AMP has anti-tumor effects on breast cancer and whether ROS generation and ER stress pathway are required for AMP-induced cell growth inhibition and apoptosis. We found that AMP treatment suppressed cell growth and induced apoptosis in breast cancer cell lines MCF-7 and MDA-MB-231. Moreover, AMP-induced cell growth inhibition and apoptosis were mediated by ROS generation and ER stress pathway. Our results will therefore lead to the development of AMP as an attractive therapeutic drug for breast cancer.

## Results

### AMP induces cell growth inhibition and apoptosis in human breast cancer cells

**Figure 1:**
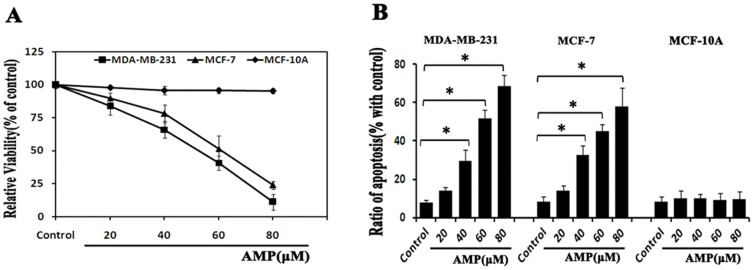
AMP inhibits cell growth and induces apoptosis in breast cancer cells. (A) Dose-dependent effects of Ampelopsin (AMP) on cell viability. MCF-10A, MDA-MB-231 and MCF-7 cells were treated with different concentrations of AMP (20, 40, 60, 80 µM) for 24-h, respectively, and cell viability was measured by CCK-8 assay. (B) Dose-dependent effects of AMP on apoptosis. After treatment with the indicated concentrations of AMP for 24-h, apoptosis was assessed using Annexin V-FITC/PI staining. All data shown are representative of three independent experiments, *P<0.05.

To investigate whether AMP has an anti-tumor role in breast cancer, the CCK-8 assay was adopted to evaluate the cytotoxic effects of AMP on estrogen receptor- positive and negative breast cancer cells lines MCF-7 and MDA-MB-231, as well as human normal breast epithelial cells MCF-10A, respectively. Notably, after treatment with 20, 40, 60 and 80 µM AMP for 24-h, there were a dose-dependent inhibition of cell viability in both breast cancer cell lines, but had no effects on human normal breast epithelial MCF-10A cells (**Figure 1A**). Because of reduction of MDA-MB-231 cell viability seemed to be more pronounced than that of MCF-7 cells, it is possible that AMP-induced growth inhibition in breast cancer cells was correlated with the estrogen receptor status. To test this prediction, we exposed estrogen receptor-positive MCF-7 cells to anti-estrogen agent ICI 182780, and then evaluated the subsequent cell viability induced by AMP treatment. However, there had no significant changes of cell viability between MCF-7 cells pre-treated with ICI 182780 for 2-h prior to AMP treatment and those treated with AMP alone (data not shown). Our studies demonstrate that AMP can induce growth inhibition of breast cancer cells, and its growth inhibitory effect is independent of estrogen receptor status.

We next assessed whether the growth inhibitory effect of AMP on breast cancer cells was correlated with increased apoptosis. After treatment with 20, 40, 60 and 80 µM AMP for 24-h, cell apoptosis was measured. In our study, AMP dose-dependently increased the percentage of apoptotic cells in both MCF-7 and MDA-MB-231 cells, whereas had little impact on apoptosis in MCF-10A cells (**Figure 1B**), suggesting that AMP induced growth inhibition of breast cancer cells, at least in part, by induction of apoptosis.

In summary, our results suggest that AMP possesses anti-cancer effects on breast cancer in vitro, and has no cytotoxic effects on human normal breast epithelial cells. Furthermore, anti-breast cancer effects of AMP are independent of estrogen receptor status of breast cancer cells.

### AMP triggers ROS generation in breast cancer cells

**Figure 2:**
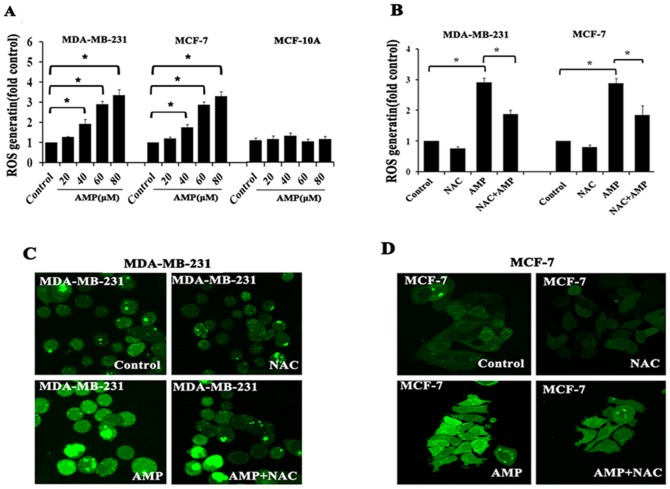
AMP triggers ROS generation in breast cancer cells. (A) Dose- dependent effects of Ampelopsin (AMP) on cellular ROS levels. MDA-MB-231, MCF-7 and MCF-10A cells were treated with the indicated concentrations of AMP for 24-h, and cellular ROS levels were assessed using 10 µM DCFH-DA, with the resulting fluorescence read on a fluorescence microplate reader. (B) The effects of N-acetyl-L-cysteine (NAC) on AMP-induced ROS generation. After pre-treatment with 5 mM NAC for 2-h, followed by treatment with 60 µM AMP for 24-h, cellular ROS levels were measured. (C) And (D) Representative fluorescence microscopy images of MDA-MB-231 and MCF-7 cells, respectively. Cells were treated as described in (A) and visualized by confocal microscopy. The results of A and B represent the mean±S.E.M. of three independent experiments, *P<0.05.

Many studies have confirmed that the anti-cancer effects of certain flavonoids are closely related to trigger ROS generation, and ROS have been considered as a potential target for anti-tumor candidates [24]–[26]. As one of flavonoids, we predicate AMP also can induce the generation of ROS in breast cancer cells. Consistent with our hypothesis, after treatment with AMP (20,40,60 and 80 µM) for 24-h, intracellular ROS level was assessed using a 2′, 7′- dichlorofluorescin diacetate (DCFH-DA) probe, and quantified using a fluorescence microplate reader or imaged by fluorescence microscopy. Data revealed that AMP dose-dependently increased ROS generation in MCF-7 and MDA-MB-231 cells, and ROS levels were increased 1.4- to 4.5-fold compared with control group, whereas AMP had little impact on ROS generation in human normal breast epithelial MCF-10A cells (**Figure 2A, 2C, 2D**). Conversely, pretreatment with the ROS inhibitor N-acetyl-L-cysteine (NAC, 5 mM) 2-h prior to AMP treatment, AMP-induced ROS generation was markedly attenuated in both breast cancer cells (**Figure 2B**). These results demonstrate that AMP treatment triggers ROS production in breast cancer cells, which is similar to other flavonoids.

### ROS are required for AMP-induced cell growth inhibition and apoptosis

**Figure 3:**
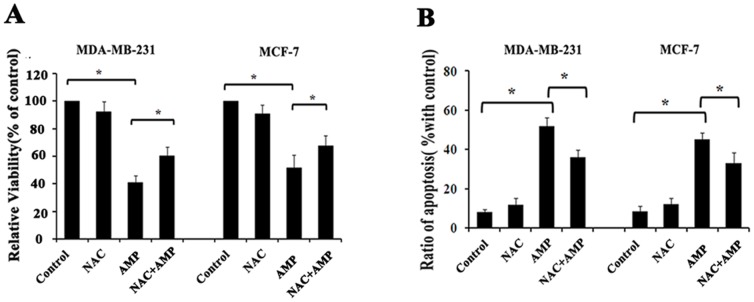
Anti-breast cancer effects of AMP are dependent on ROS. (A) The effects of NAC on AMP-induced cell growth inhibition. After pre-treatment with 5 mM NAC for 2-h, followed by treatment with 60 µM AMP for 24-h, cell viability was assessed by CCK-8 assay. (B) The effects of NAC on AMP-induced apoptosis. After cells were treated as described in (A), cell apoptosis was measured by Annexin V-FITC/PI staining. All data shown represent the mean±S.E.M. of three independent experiments, *P<0.05.

Quite a lot of reports have revealed that oxidative stress plays a role in the anti- cancer activities of chemotherapeutic drugs, and ROS generation has been confirmed to be closely related to trigger apoptosis [27], [28]. We therefore next determined whether ROS generation is implicated in the growth inhibition and pro-apoptotic effects of AMP. The ROS scavenger NAC (5 mM) was added 2-h before AMP administration (60 µM), and then evaluated the subsequent cell viability induced by AMP treatment. As expected, we found that pre-treatment with NAC significantly inhibited AMP-induced growth inhibition in MDA-MB-231 and MCF-7 cells (**Figure 3A**). As well, similar results were observed in AMP-induced apoptosis. NAC treatment markedly decreased AMP-induced apoptosis in both breast cancer cells (**Figure 3B**). Collectively, these data reveal that AMP-induced cell growth inhibition and apoptosis in breast cancer cells are, at least in part, dependent on ROS production.

### Ampelopsin activates ER stress in breast cancer cells

**Figure 4:**
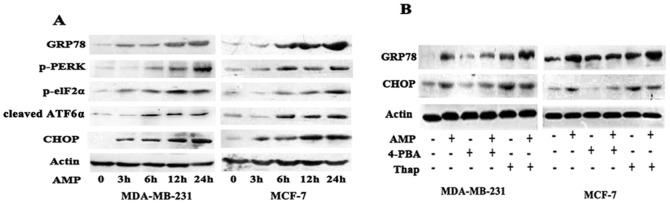
AMP activates ER stress in breast cancer cells. (A) Time-dependent effects of Ampelopsin (AMP) on ER stress-associated proteins. MDA-MB-231 and MCF-7 cells were treated with 60 µM AMP for indicated time, and the protein expression levels of GRP78, p-PERK, p-elF2α, cleaved ATF6α, and CHOP were assessed by western blotting. (B) The effect of ER stress inhibitors or activators on AMP-induced ER stress in breast cancer cells. After pre-treatment with 2 mM 4-Phenylbutyric acid (4-PBA) or 150 nM thapsigargin (Thap) for 2-h, respectively, followed by treatment with 60 µM AMP for 24-h, the expression of GRP78 and CHOP were assessed by western blotting. All results are representative western blots of three independent experiments with similar results.

The accumulation of misfolded or unfolded proteins in response to ROS-mediated oxidative stress can trigger endoplasmic reticulum (ER) stress as an adaptive cellular response [25], [29]. ER stress can be characterized by an increase in ER stress-associated molecules including GRP78, p-PERK, p-elF2α, and ATF6α [30], [31]. In addition, GRP78 and CHOP have been considered as two vital proteins of ER stress response [31], [32].We next determined whether AMP regulated the expression of ER stress-associated proteins. Data revealed that AMP exposure time-dependently increased the expression of GRP78, p-PERK, p-elF2α, cleaved ATF6α, and CHOP in both breast cancer cell lines MCF-7 and MDA-MB-231 (**Figure 4A**). After pre-treated with 2 mM 4-phenylbutyric acid (4-PBA), an ER stress inhibitor, or 150 nM thapsigargin (Thap), an ER stress inducer, respectively, for 2-h before 60 µM AMP treatment, and then expression of GRP78 and CHOP were examined. 4-PBA treatment markedly down-regulated AMP-induced expression of GRP78 and CHOP (**Figure 4B**), but Thap treatment obviously promoted AMP-induced GRP78 and CHOP expression in both breast cancer cells (**Figure 4B**). These results show that AMP can activate ER stress, evidenced by up-regulating the expression of ER stress-associated proteins.

### ER stress is involved in Ampelopsin-induced cell growth inhibition and apoptosis in human breast cancer cells

**Figure 5:**
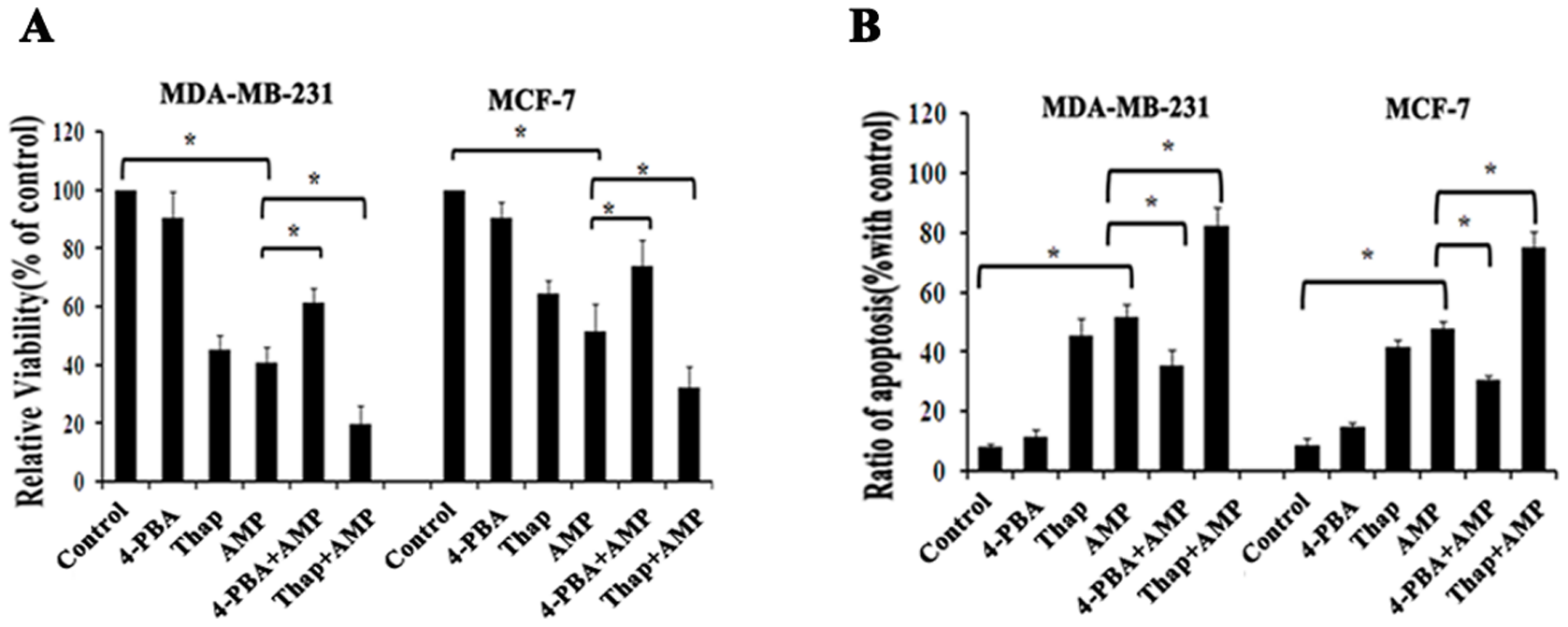
ER stress is involved in anti-tumor effects of AMP against breast cancer cells. (A) The effects of ER stress inhibitors or activators on AMP-induced cell growth inhibition in breast cancer cells. After pre-treatment with 2 mM 4-Phenylbutyric acid (4-PBA) or 150 nM thapsigargin (Thap) for 2-h, respectively, followed by treatment with 60 µM AMP for 24-h, cell viability was measured. (B) The effects of ER stress inhibitors or activators on AMP-induced apoptosis. After cells were treated as described in (A), cell apoptosis was measured. All data shown represent the mean±S.E.M. of three independent experiments *P<0.05.

Recently, ER stress has been considered as a vital regulator of various cellular pathological processes, including cancer cell death pathways in response to anti- cancer drugs [31], [33]. To confirm the role of ER stress in cell death induced by AMP, MCF-7 and MDA-MB-231 cells were pre-treated with 2 mM 4-PBA, and 150 nM Thap for 2-h before 60 µM AMP treatment, and then cell viability and cell apoptosis were examined, respectively. We found that ER stress inhibitor 4-PBA significantly attenuated AMP-induced cell growth inhibition and apoptosis in both breast cancer cells, but ER stress inducer thapsigargin obviously promoted AMP- induced cell growth inhibition and apoptosis (**Figure 5A, 5B**). These results suggest that activating ER stress signaling pathways is closely related to AMP-induced cell growth inhibition and apoptosis in breast cancer.

### PERK-CHOP pathway is mainly involved in ER stress-mediated apoptosis in human breast cancer cells induced by AMP

**Figure 6:**
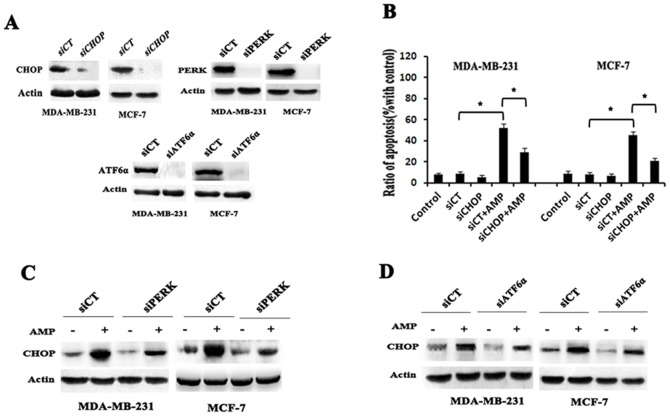
PERK-CHOP pathway is mainly involved in ER stress- mediated apoptosis in human breast cancer cells induced by AMP. (A) The inhibition efficiency of siRNAs against CHOP, ATF6α and PERK, respectively. Cells were transfected with siRNAs targeting CHOP, ATF6α and PERK (100 nM each) for 24-h, and the protein levels of CHOP, ATF6α and PERK was determined by western blot, respectively. (B) The effects of CHOP siRNA on AMP-induced cell apoptosis. Cells were transfected with control (siCT) or 100 nM CHOP siRNA (siCHOP), followed by treatment with 60 µM AMP for 24-h, then cell apoptosis was measured. The data shown represent the mean±S.E.M. of three independent experiments *P<0.05. (C) And (D) The effects of PERK or ATF6α siRNA on AMP-induced CHOP expression. Cells were transfected with control (siCT) or 100 nM PERK siRNA (siPERK) or ATF6α siRNA (siATF6α), followed by treatment with 60 µM AMP for 24-h, then the protein levels of CHOP were determined by western blot. All results are representative western blots of three independent experiments with similar results.

All three arms of ER stress signaling pathway initiated by ATF6, IRE1α and PERK respectively are capable of inducing CHOP, which plays an important role in ER stress- mediated apoptosis. To confirm the role of CHOP in AMP-induced apoptosis of breast cancer cells, we shut down of CHOP using RNA interference, and then examined the change of cell apoptosis induced by AMP. As expected, we found CHOP knockdown significantly attenuated AMP-induced apoptosis in both breast cancer cells (Figure 6B). Furthermore, we assessed the effects of ATF6α and PERK silencing by RNA interference on AMP-induced CHOP expression, respectively. Our study found that the expression of CHOP induced by AMP could be down-regulated by knockdown of PERK or ATF6αin both MCF-7 and MDA-MB-231 cells. Interestingly, in our study, ATF6αknockdown had a relative weaker activity of down-regulating expression of CHOP, compared to PERK knockdown (Figure 6C, 6D). Taken above all results, it suggests that AMP induces expression of CHOP through PERK and ATF6-mediated signaling pathway in human breast cancer cells, and PERK-CHOP pathway is mainly involved in ER stress-mediated cell apoptosis induced by AMP.

### Ampelopsin induces ROS and ER stress to form a vicious cycle in breast cancer cells

**Figure 7:**
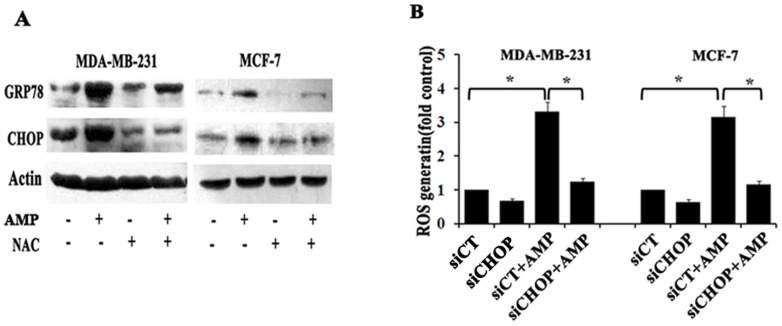
Ampelopsin induces ROS and ER stress to form a vicious cycle in breast cancer cells. (A) Effects of NAC on AMP-induced ER stress. After pre-treatment with 5 mM NAC for 2-h, followed by treatment with 60 µM AMP for 24-h, CHOP and GRP78 expression were evaluated by western blotting. (B) The effects of CHOP siRNA on AMP-induced ROS generation. Cells were transfected with control (siCT) or 100 nM CHOP siRNA (siCHOP), followed by treatment with 60 µM AMP for 24-h. Then cellular ROS levels were assessed. The results of A and C represent the mean±S.E.M. of three independent experiments *P<0.05.

To test the relationship between ROS generation and ER stress induced by AMP, we first used the ROS scavenger NAC to block ROS generation, and then examined expressions of ER stress critical proteins GRP78 and CHOP. After MCF-7 and MDA-MB-231 cells treated with 5 mM NAC 2-h before 60 µM AMP treatment, GRP78 and CHOP expressions induced by AMP were attenuated in both breast cancer cells, compared with those treated with AMP alone (**Figure 7A**), suggesting that eliminating ROS accumulation by NAC could alleviate the ER stress. Next, we aimed to examine whether ER stress was involved in regulating ROS generation triggered by AMP. We shut down CHOP by RNA interference and then examined the level of ROS production in breast cancer cells after treatment with AMP. Interestingly, the level of ROS production induced by AMP could be significantly blocked by RNA interference against CHOP in both breast cancer cells (**Figure 7B**), implying that blocking ER stress could suppress ROS production- induced by AMP. These data indicate that ROS generation induced by AMP is a powerful trigger of ER stress, and in turn, severe ER stress can enforce ROS generation. In other words, AMP-induced ROS generation and ER stress in breast cancers may form a vicious cycle.

## Discussion

Here, our study is first shown that AMP possessed anti-breast cancer capability through ROS generation and ER stress pathway. This novel finding is approved by the following evidences: (i) AMP dose-dependently decreases cell viability and induces apoptosis both in MCF-7 and MDA-MB-231 breast cancer cells without cytotoxic effect on normal human mammary epithelial cells; (ii) AMP stimulates ROS accumulation, and blocking ROS generation strongly inhibits AMP-induced growth inhibition and apoptosis; (iii) Accompany with ROS production, AMP triggers ER stress, and blocking ROS generation markedly inhibits AMP-induced ER stress. Moreover, blocking ER stress predominantly decreases AMP-induced cell death and ROS generation in both breast cancer cells.

AMP is a flavonoid, and the major bioactive constituent of *Ampelopsis grossedentata*, which has been widely used as a tea product in South China [8], [10], [11]. Recently, it was reported that AMP had anti-cancer effects in bladder carcinoma and prostate cancer [13], [14]. In the present study, we observed that AMP dose-dependently inhibited the growth of MDA-MB-231 and MCF-7 breast cancer cells, and had no cytotoxic effect on human normal mammary epithelial MCF-10A cells. These results suggest that AMP is cytotoxic to breast cancer cells but not to normal breast cells. Our study also found that AMP-induced growth suppression of breast cancer cells is independent of estrogen receptor status, supported by observations that pretreatment with ICI182780, an anti-estrogen, did not alter the sensitivity of estrogen receptor-positive MCF-7 to AMP treatment. Furthermore, we found that AMP induced apoptosis in both breast cancer cell lines in a dose-dependent manner, suggesting that the AMP-mediated cell growth inhibition may be partly owing to the induction of apoptosis. Collectively, these observations demonstrate that AMP is a prospective chemotherapeutic agent against breast cancer. However, it is necessary to further evaluate its anti-breast cancer effects in vivo studies.

ROS, which can be generated by multiple mechanisms, are highly reactive oxygen free radicals or non-radical molecules, and mainly originated from NADPH oxidase (NOX) and mitochondria [16], [20]. ROS act as important multi-faceted signaling molecules that regulate plenty of cellular pathways, and therefore play key roles in cell fate determination [16], [29], [34]. It is well known that accumulation of ROS can result in oxidative stress, impairment of cell function, and necrosis or apoptosis. A large number of experiments have demonstrated that ROS-mediated pathways play an important role in cell apoptosis induced by certain flavonoids [24]–[26]. We therefore set out to assess whether AMP-induced cell growth inhibition and apoptosis are also dependent on ROS generation. We found that AMP dose-dependently increased ROS generation, which was accompanied by increased apoptosis in both MDA-MB-231 and MCF-7 breast cancer cells, but it had little impact on ROS generation in human normal mammary epithelial MCF-10A cells. Conversely, the inhibition of ROS by pre-treatment with the ROS scavenger NAC decreased not only intracellular ROS levels but also growth inhibition and apoptosis induced by AMP, suggesting that the anti-breast cancer effects of AMP are partially dependent on ROS production. In conclusion, AMP triggers ROS generation, and ROS generation, at least partially, is required for AMP-induced cell growth inhibition and apoptosis in breast cancer cells.

It is well known that high level of ROS accumulation can cause proteins damage which further lead to endoplasmic reticulum (ER) stress response. ER plays a critical role in cellular protein folding and modification [21], [22], [25]. A number of severe conditions can result in the accumulation and aggregation of unfolded and/or misfolded proteins in the ER lumen, and then activate an ER stress response termed the unfolded protein response (UPR) [35], [36]. The UPR is initiated by three ER-resident transmembrane proteins known as sensors of ER stress, PKR-like ER kinase (PERK), activating transcription factor 6 (ATF6), and inositol-requiring kinase 1 (IRE1)[36], [37]. In addition, ER stress is considered as a vital regulator of various cellular pathological processes, including cancer cell death pathways in response to anti-cancer drugs [21], [22], [33]. Mounting data indicate that ER stress plays an important role in the regulation of apoptosis [3], [25], [38]. The CAAT/enhancer binding protein homologous protein (CHOP) has been reported to be a crucial ER stress responsive factor that executes apoptosis, which can be induced and up-regulated by all three arms of the UPR signaling pathways [39], [40]. This study provides important evidence that AMP activates ER stress signaling pathways in both MCF-7 and MDA-MB-231 cells, and that the induction of ER stress is implicated in AMP-induced cell growth inhibition and apoptosis. Specifically, we demonstrated that (i) AMP induced the up-regulation of GRP78, p-PERK, p-elF2α, and cleaved ATF6α, all of which mediate ER stress in both breast cancer cells; (ii) AMP also increased the expression of CHOP, which is an important apoptotic inducer. Blocking CHOP by RNA interference markedly decreased AMP-induced cell apoptosis, suggesting induced apoptosis is mediated, at least in part, by ER stress-induced up-regulation of CHOP; (iii) Blocking ER stress by ER stress inhibitor 4-PBA not only effectively decreased AMP-induced GRP78 and CHOP expression, but also significantly decreased AMP-induced apoptosis. Meanwhile, ATF6αor PERK knockdown down-regulated AMP-induced CHOP expression, and ATF6αknockdown had a relative weaker activity of down-regulating expression of CHOP, compared to PERK knockdown. Collectively, these results indicate that AMP-induced growth inhibition and apoptosis in breast cancer cells are partially dependent on ER stress through up-regulation of CHOP expression via activating two branches of unfolded protein response (UPR), PERK and ATF6, and that PERK-CHOP pathway may be mainly involved in ER stress-mediated apoptosis induced by AMP.

Several studies have shown that oxidative stress and ER stress are closely related events. Excessive levels of ROS can efficiently induce protein misfolding in the ER, and then activate the ER stress [29], [38], [39]. Conversely, prolonged ER stress can induce the generation of ROS [29], [41]. In our study, AMP dose-dependently increased ROS generation in both MCF-7 and MDA-MB-231 breast cancer cells; blocking ROS production by ROS scavenger NAC dramatically attenuated AMP-induced GRP78 and CHOP expression, which are two ER stress markers, suggesting that ROS formation argued for an upstream event of ER-stress induced by AMP. Interestingly, blocking UPR pathway by using RNA interference against CHOP obviously inhibited AMP-induced ROS generation, suggesting that ROS production induced by AMP could occur downstream the UPR activation and ER stress which in turn increases ROS production. Although the exact underlying mechanisms remain unknown, based on our results, we support the idea that oxidative stress and ER stress may form a vicious cycle [42]. Elucidating the correlation between ROS generation and UPR represents a major area for our future research.

In summary, we demonstrated that cell growth inhibition and apoptosis could occur simultaneously in breast cancer cells exposed to AMP, and that these changes were partially mediated by ROS generation and ER stress pathway. These findings may be helpful to the development of AMP into a chemotherapeutic drug for breast cancer. However, there are two limitations to this study. First is that the downstream signaling pathways involved in ROS-dependent ER stress were not fully elucidated, which would have further revealed the molecular mechanism of anti-breast cancer of AMP. Additionally, the anti-breast cancer effects of AMP were not studied in vivo. However, appended studies to address both of these issues are ongoing.

## Materials and Methods

### Antibodies and Reagents

Ampelopsin (AMP) were bought form Chengdu Must Bio-technology CO., LTD (MSAT-12013108, HPL≥98%).N-acetyl-L-cysteine (NAC, A9165), 4-Phenylbutyric acid (4-PBA, P21005) and thapsigargin (Thap, T9033) were purchased from Sigma- Aldrich. Antibodies against GRP78 (sc-1051), p-PERK (sc-3257), PERK (sc-13073), ATF6α (sc-22799) and CHOP/GADD153 (sc-575) were obtained from Santa Cruz Biotechnology (Santa Cruz, CA). Antibody against p-elf2α(#4688) was obtained from Cell signaling Technology. And antibody against Actin (TA-09) was obtained from Zhongshan Jinqiao Biotechnology Co. (Beijing, China). Lipofectamine™ 2000 transfection reagent was purchased from Invitrogen (Life Technologies, NY, USA). The Annexin V-FITC Apoptosis Detection kit was purchased from BestBio (Shanghai, China). Cell Counting Kit-8 (CCK-8) was purchased from Dojindo Laboratories (Kumamoto, Japan). Probes 2′, 7′- dichlorofluorescin diacetate (DCFH-DA) was purchased from Beyotime (Shanghai, China).

### Cell Culture and treatment

Human normal breast epithelial cell MCF-10A, breast cancer cell lines MCF-7 and MDA-MB-231 were purchased from Institute of Biochemistry and Cell Biology, Chinese Academy of Sciences (Shanghai, China). MCF-10A were propagated in DMEM/F12 media supplemented with mitogenic additives including 100 ng/ml cholera enterotoxin, 10 mg/ml insulin, 0.5 mg/ml hydrocoritisol, 20 ng/ml epidermal growth factor, and 5% horse serum. MCF-7 and MDA-MB-231 cells were propagated in DMEM media supplemented with 10% fetal bovine serum, 100 U/ml penicillin and 100 µg/ml streptomycin in a humidified atmosphere of 95% air with 5% CO_2_ at 37°C. Cells in mid-logarithmic growth were used for the following experiments. Stock solution of AMP was prepared in DMSO and an equal volume of DMSO (final concentration 0.1%) was added to the control. As well, when reached 75% confluences, cells were treated with the indicated concentration of AMP. When indicated, NAC (5 mM), 4-PBA (2 mM), and Thap (150 nM) were added 2 h before AMP administration.

### Cell Viability Measurement

The Cell Counting Kit-8 (CCK-8) was used for measure cell viability. Briefly, according to the manufacturer's directions, cells were cultured in a 96-well plate and exposed to various treatments as indicated for 24-h. The control group was treated with 0.1% DMSO. Then, 10 µl CCK-8 was added to each well, and the plate was incubated at 37°C for 2-h. Optical density (OD) values were assessed at 450 nm with the Infinite™ M200 Microplate Reader (Tecan, Männedorf, Switzerland). Cell viability was expressed as percentage of the vehicle controls. All experiments were performed in triplicate and repeated three times.

### Annexin V-FITC/PI assay

Annexin V/P staining was used to quantify the effect of AMP on apoptosis with Annexin V-FITC Apoptosis Detection kit. Following the manufacture's protocol and quantified by flow cytometry. Briefly, cells were cultured overnight in 6-well plates and then exposed to various treatments as indicated for 24-h. After washing with ice-cold PBS, the cells were detached in trypsin and centrifuged (5 min, 4°C, 2000 rmp), followed by resuspended the cells in 200 µl PBS.The cells were centrifuged again, and resuspended in 200 µl 1×Annexin bingding buffer. Then, the cells were incubated with Annexin V-FITC (2.5 µl) and propidium iodide (5 µl) for 15 min at room temperature. Samples were then analysed for apoptosis by a FACScan flow cytometer (Becton Dickinson, Franklin Lakes, NJ).

### Reactive oxygen species (ROS) assay

The probe of DCFH-DA was used for assess the production of ROS. Briefly, according to the manufacturer's directions, for detection of ROS, cells were grown on glass coverslides. When cells reached 75% confluence, they were exposed to different treatments for 24-h. After washing with serum-free medium, cells were then incubated with DCFH-DA (10 µM) at 37°C for 30 min in the dark. At the end of the incubation process, cells were washed again with serum-free medium and imaged by confocal microscope. To quantitate ROS levels, cells were seeded on a 96-well plate and treated as described above. Relative fluorescence was detected with an Infinite™ M200 Microplate Reader at the excitation and emission wavelengths of 485 and 528 nm, respectively, for three times. Cellular fluorescence intensity was expressed as a multiple of the level in the control groups.

### siRNA assay

siRNAs for CHOP/GADD153 (human, sc-35437), PERK (human, sc-36213) and ATF-6α (human, sc-37699) were purchased from Santa Cruz Biotechnology, as well control siRNA. According to the manufacturer's protocol, cells were transfected with 100 nM siRNA, then LF2000-containing medium was replaced with fresh DMEM media for 24-h. After that, cells were treated with indicated treatments and were used for further experiments.

### Western Blot

After treatment, about 1×10^7^ cells were harvested, washed twice with ice-cold PBS, and lysed with RIPA buffer at 4°C for 60 min. Cell lysates were centrifuged for 12,000 g×30 min at 4°C. Protein concentration was determined using the Bio-Rad DC*^TM^* Protein Assay Kit. About thirty to fifty micrograms of protein were separated using SDS-PAGE and transferred to PVDF membranes. The membranes were blocked in 5% skim dry milk (2-h), rinsed, incubated with primary antibodies (diluted 1∶1000) overnight at 4°C, followed by HRP-conjugated secondary antibodies (diluted 1∶5000) for 1.5- h at room temperature. The proteins were visualized by ECL exposure to X-ray film.

### Statistical Analysis

All of the experimental data are expressed as the mean±S.E.M, and each experiment was performed at least three times. The statistical analysis was performed by *t*-text and one-way analysis of variance, using SPSS 13.0 (SPSS Inc, Chicago, Ill). *P*<0.05 was considered as statistically significant, and Turkey-*K*ramer was applied as post-hoc test if *P*<0.05.
